# Progress and prospects of early detection in lung cancer

**DOI:** 10.1098/rsob.170070

**Published:** 2017-09-06

**Authors:** Sean Blandin Knight, Phil A. Crosbie, Haval Balata, Jakub Chudziak, Tracy Hussell, Caroline Dive

**Affiliations:** 1 North West Lung Centre, University Hospital South Manchester, Manchester, UK; 2 Cancer Research UK Lung Cancer Centre of Excellence at Manchester and University College London, UK; 3 Clinical and Experimental Pharmacology Group, Cancer Research UK Manchester Institute, University of Manchester, Manchester, UK; 4 Manchester Collaborative Centre for Inflammation Research, University of Manchester, Manchester, UK

**Keywords:** lung cancer, low-dose CT screening, NLST trial

## Abstract

Lung cancer is the leading cause of cancer-related death in the world. It is broadly divided into small cell (SCLC, approx. 15% cases) and non-small cell lung cancer (NSCLC, approx. 85% cases). The main histological subtypes of NSCLC are adenocarcinoma and squamous cell carcinoma, with the presence of specific DNA mutations allowing further molecular stratification. If identified at an early stage, surgical resection of NSCLC offers a favourable prognosis, with published case series reporting 5-year survival rates of up to 70% for small, localized tumours (stage I). However, most patients (approx. 75%) have advanced disease at the time of diagnosis (stage III/IV) and despite significant developments in the oncological management of late stage lung cancer over recent years, survival remains poor. In 2014, the UK Office for National Statistics reported that patients diagnosed with distant metastatic disease (stage IV) had a 1-year survival rate of just 15–19% compared with 81–85% for stage I.

## 1. Introduction

Lung cancer is the leading cause of cancer-related death in the world [1]. It is broadly divided into small cell (SCLC, approx. 15% cases) and non-small cell lung cancer (NSCLC, approx. 85% cases). The main histological subtypes of NSCLC are adenocarcinoma and squamous cell carcinoma, with the presence of specific DNA mutations allowing further molecular stratification [2]. If identified at an early stage, surgical resection of NSCLC offers a favourable prognosis, with 5-year survival rates of 70–90% for small, localized tumours (stage I) [3–5]. However, most patients (approx. 75%) have advanced disease at the time of diagnosis (stage III/IV) [6] and despite significant developments in the oncological management of late stage lung cancer over recent years, survival remains poor. In 2014, the UK Office for National Statistics reported that patients diagnosed with distant metastatic disease (stage IV) had a 1-year survival rate of just 15–19% compared with 81–85% for stage I [7].

SCLC is more aggressive than NSCLC, with a prognosis that is even worse—overall 5-year survival is around 5%. Early dissemination is characteristic and consequently more than 90% of patients present with locally advanced or distant metastatic disease (stage III/IV) [6]. The window for radical treatment is narrow [8], but on the rare occasion patients are identified with stage I disease, surgery still appears to be beneficial. One study reported 5-year survival rates of 40% for resection alone and 52% with adjuvant chemotherapy/radiotherapy [9]. A recent propensity-matched analysis compared surgery combined with chemotherapy and radiotherapy to chemotherapy and radiotherapy alone for stage I SCLC, and found significant improvement in survival in those who had surgery [10]. Hence, for both SCLC and NSCLC there is a clear rationale for detection of early stage disease. This review will summarize current clinical and experimental studies to highlight progress as well as some of the challenges in early detection of lung cancer. There are many possible means of early detection of lung cancer and those discussed here are non-exhaustive.

## 2. Biology of early lung cancer

A potential additional avenue for early disease biomarker discovery may lie in understanding the changes in the lung environment itself during the initial stages of cancer development. The lung is continually exposed to the external environment and functions under extreme changes in local pressure during the ventilation process. As such, it has evolved into a highly specialized structure. The branching conducting airways are lined with a complex, pseudostratified epithelium composed predominantly of ciliated cells, but also containing various rare populations of secretory cells that are capable of serving as progenitor cells during airway repair following injury [11–13]. In contrast to the complexity of the conducting airways, the alveoli are lined with only two cell types—squamous type I alveolar epithelial cells, which account for approximately 90% of alveolar coverage and are responsible for capillary interaction, and cuboidal type II alveolar epithelial cells, which are responsible for secreting lipids and proteins that reduce surface tension during ventilation, and may also act as type I cell precursors during alveolar repair [14].

The results of lineage tracing experiments point, not unreasonably, to cells responsible for airway repair as the initiating cells of tumours in the lung. Although the cell of origin of human SCLC is yet to be formally identified, the expression of neuroendocrine markers, along with data obtained from mouse models, point to neuroendocrine cells (one of the rare secretory cell populations in the conducting airways) as the starting point for SCLC [15–18]. In the case of NSCLC, the major cell of origin for *Kras*-driven adenocarcinomas is in the alveoli, namely alveolar type II epithelial cells, while the cell of origin for squamous cell carcinoma is yet to be confirmed [19].

The association between the presence of chronic, unresolved inflammation and the increased risk of cancer development is well described [20,21]. Although only approximately 20% of cancers are related to chronic inflammation, innate immune cells and mediators are found in the majority of human malignancies [20,22]. One of the reasons for this is the induction of inflammatory pathways in both pre-malignant and malignant cells caused by oncogenic changes, meaning inflammation can lead to cancer, but cancer can also lead to inflammation [20]. The tumour microenvironment coordinates pro-inflammatory responses through both inflammatory cells as well as mediators (cytokines, chemokines and prostaglandins), all of which are capable of acting in an autocrine and paracrine fashion, affecting both malignant and non-malignant cells [23].

The inflammatory microenvironment consists of tumour-infiltrating inflammatory cells, tumour-associated fibroblasts and endothelial progenitor cells [21,24–26]. Both tumour and host cells, including stromal, endothelial and immune cells, release cytokines and chemokines within this microenvironment, enabling the coordination of a self-limiting immune response [20]. Key factors in this setting include tumour necrosis factor-α (TNF-α), interleukins 6, 1α and 8 (IL-6, IL-1α, IL-8), the inflammatory chemokine CCL2, as well as the CXCL12–CXCR4 signalling cascade. Between them they are responsible for a diverse range of effects, such as generation of inflammation-associated immune responses and recruitment of inflammatory cells, promotion of cell growth, survival, invasion and angiogenesis [20,27–37].

The interactions described above are in no way an exhaustive list, with more investigation needed to fully elucidate the interactions between tumour, environment and immune system during the early stages of cancer development. Further investigation of these events and parallel development of preclinical models of early disease in which to study them has the potential to yield novel biomarkers for early detection of lung cancer. However, this remains challenging and is under researched.

## 3. Lung cancer screening

The efficacy of lung cancer screening, using either chest X-ray (CXR) or more recently computed tomography (CT), with or without additional adjuncts such as sputum cytology, has been investigated in numerous studies in asymptomatic at risk populations. Although earlier disease stage shift is an important outcome, the gold standard measure of screening efficacy is mortality reduction. This is to reduce the impact of lead time bias, where earlier detection of a cancer can result in an apparent improvement in survival without changing the eventual disease course (i.e. time of death remains the same).

Optimal delivery of lung cancer screening requires the targeting of those individuals most at risk. In this respect lung screening differs from other population-based screening programmes such as bowel, breast and cervix where all individuals of a certain age and sex are eligible. However, it is apparent from lung cancer screening trials that a significant participation bias exists, with those most at risk least likely to participate [38]; in both the National Lung Screening Trial (NLST) and the UK Lung Cancer Pilot Screening Trial (UKLS), current smoking, older age and lower socioeconomic status were associated with reduced uptake [39–41]. How to engage this so-called ‘hard-to-reach’ group is of fundamental importance for lung cancer screening implementation. The reasons given for non-participation in UKLS included travel, cost and inconvenience. One approach to improve service accessibility and overcome these practical barriers is to move screening ‘closer to home’ [39,42]. Community-based screening programmes, which use mobile CT scanners, may address some of these issues and improve participation. Although no study has directly addressed this, compliance in trials using mobile CT scanners is good [43,44] and this type of approach has been effective in breast cancer screening [45]. We are currently evaluating a community-based approach to lung cancer screening in areas of high deprivation and lung cancer incidence in Manchester. Emotional barriers, such as fear, anxiety and avoidance, are also important factors [39]. Engagement of at-risk groups is essential to overcome this; approaches may include primary care endorsed invitational material, low-impact information leaflets that reduce the emphasis on ‘cancer’ and focus more on the health check aspect of screening and cancer champions who provide positive encouragement for people to attend.

### 3.1. Chest X-ray screening

Several randomized control trials in the 1970s used plain CXR and sputum cytology to detect early lung cancer. In the Mayo Clinic trial, patients were randomized to an intensive screening group where sputum cytology and CXRs were checked every 4 months or a control group where the standard advice of yearly screens was given. There was no difference in mortality between 4 monthly and yearly screens [46] and this remained the case even after extended follow up (median 20.5 years) [47]. Although more resectable tumours were identified in the intensively screened group, the number of unresectable tumours was equal between the two groups and not higher in the control group as one would expect if screening were beneficial [48]. More than 80% of lung cancers detected by sputum cytology alone were amenable to resection, but only accounted for 20% of diagnoses [49]. Two studies, at Johns Hopkins and Memorial Sloan-Kettering cancer centres, including approximately 10 000 patients each, compared plain CXR with or without sputum cytology. Of the lung cancers that developed in the patient cohorts with dual screening, about 20% were detected by cytology alone—almost exclusively early stage squamous cell carcinomas. However, there was no difference in mortality by addition of cytology screening [50,51].

More recently the Prostate, Lung, Colorectal and Ovarian Cancer Screening trial (PLCO) randomized 150 000 patients to either annual CXR or usual care (i.e. no screening intervention). In contrast to the Mayo Clinic, Johns Hopkins and Memorial Sloan-Kettering trials, only around 50% were current or former smokers. In agreement with previous studies, there was no mortality benefit in screening with CXR. However, there were some positive aspects for reflection; 60% of lung cancers that developed within the screening period were detected by the screening CXR (rather than ‘interval’ cancers), and of these, half were stage I disease [52].

### 3.2. Low-dose CT screening

CT delivers more detailed images of the chest than CXR and, therefore, is more useful for diagnosing cancer. However, it is generally accepted that the radiation dose, which is about 100 times higher than CXR [53], is too high for the benefits of early diagnosis to outweigh the risks of radiation exposure. Thus it was not until CT was validated at lower radiation doses that there was a renewed appetite for lung cancer screening [54]. This ‘low-dose CT’ (LDCT) has 22% of the effective radiation dose of a standard CT [55]. Screening with LDCT still poses a risk of radiation induced cancers, recently estimated at 4 per 10 000 patients screened in a trial in Milan, although this included the radiation dose of follow up PET CTs for positive LDCT scans, which carry a significantly higher radiation dose [56]. Balancing this risk against the benefits of screening, the authors of this study have suggested that LDCT can be considered safe, but suggested altering protocols to reduce PET CTs in screening trials to mitigate risks of radiation exposure.

There have now been several LDCT trials, all have focused on at-risk populations, defined according to age and smoking exposure (generally 20–30 pack years). Two Italian studies (DANTE, *n* = 2472 [57,58] and MILD, *n* = 4099 [59]) and a Danish study (DLCST, *n* = 4104 [60]) randomized individuals to LDCT screening or a control arm, which at most included yearly medical reviews. LDCT screening had no impact on lung cancer mortality in any of these studies, despite the increased detection of early stage disease. In addition, there was no difference in the number of late stage cancers detected in the DANTE and DLCST trials where this comparison is presented.

Given the increased diagnosis of lung cancer by LDCT, but apparent lack of a mortality benefit, it was postulated that these studies were underpowered. To address this, the NLST randomized a much larger population (*n* = 53 454 participants) at risk of lung cancer (55–74 years, ≥30 pack years, smoked within 15 years) to either annual LDCT or CXR over three years. This seminal study demonstrated a significant reduction in lung cancer specific (20%) and all-cause (6.7%) mortality in the LDCT arm [61]. Furthermore, there was also a reduction in late-stage diagnosis, indicating successful identification and management of early stage disease. As a direct consequence of NLST, the US Preventative Services Task Force (USPSTF) published recommendations supporting LDCT screening and advised extending the upper age limit to 80 years [62].

Although successful, there are several issues raised by the NLST that will be informative for future lung cancer screening. There were 231 more stage 1A and 1B cancers diagnosed in the LDCT group compared with control, 93% of which were surgically resected, yet overall there were only 79 fewer lung cancer-related deaths in the LDCT group. This may be due to relatively high rates of disease recurrence even after ‘successful’ surgery for early stage disease. It is estimated to affect 30–50% of patients, most commonly at sites distant to the resected primary tumour (e.g. brain, bone and liver, where outcomes are very poor) [63]. One explanation for the higher detection rate in the LDCT arm is over-diagnosis, which is estimated to account for 18% of cancers in NLST. Over-diagnosis is due to the investigation and treatment of indolent lung cancers that would never have become clinically significant [64]. This is an increasingly recognized phenomenon most often caused by treatment of adenocarcinomas with a lepidic growth pattern (e.g. adenocarcinoma *in situ* or minimally invasive adenocarcinomas). However, in a recent LDCT randomised trial lower mortality rates were observed in the LDCT group (although not reaching statistical significance) without evidence of over-diagnosis [65]. Interestingly, SCLCs were not detected at earlier stages by LDCT.

Another challenge was the high false-positive rate of LDCT, which was 96%. This was in part due to the policy to refer any nodule more than 4 mm in diameter for further investigation. This has been addressed in the ongoing NELSON trial, comparing LDCT to no screening, where a volumetric analysis of nodules significantly reduced the false-positive rate [66]. In the UKLS trial, a structured nodule management protocol reduced the false-positive rate to 3.6%, although 23% of participants required additional interval scanning [67]. The NELSON study has also provided an interesting comparison of screen detected and interval cancers in the LDCT group, where 69% of cancers that emerged outside of screening were late stage versus 5% of screen-detected cancers [68].

### 3.3. Selecting the target population

Lung cancer screening requires specific targeting of those at most risk to be effective. For example, there is no evidence of benefit in screening never-smokers. Further analysis of the NLST population, after stratification by lung cancer risk, demonstrated that even in current or former smokers there was a marked difference in lung cancer detection rates. Almost no lung cancers were detected in the lowest risk quintile compared with 88% in the highest three risk quintiles [69]. In addition, only a fraction of individuals diagnosed with lung cancer, in routine clinical practice, would be eligible for screening by NLST criteria. The application of a more precise risk calculator [70] to the NLST population demonstrated that the mortality benefit of LDCT was consistent for individuals with a lung cancer risk score of ≥1.51% over 6 years, or above the 65th centile of risk, thus defining a more precise threshold for screening [71]. Moreover, application of risk calculators is likely to improve the cost-effectiveness of screening [72].

## 4. Bronchoscopy

Bronchoscopy has an established role in the diagnosis and, more recently, with the introduction of endobronchial ultrasound, the nodal staging of lung cancer [73,74]. With serious complication rates of about 1% [75] and the ability to be performed safely in an outpatient setting [76], bronchoscopy could play a role in early detection of lung cancer. However, sensitivity for detection of lung cancer in patients for whom this diagnosis is suspected is variable, ranging from 34% to 88% depending on size and position of tumour [77]. If applied in a screening context to patients at risk of lung cancer, but without suspicious radiological imaging per se, sensitivity may be even lower. In an effort to improve the sensitivity of bronchoscopy in lung cancer diagnosis, Spira *et al.* [78] analysed RNA expression of histologically normal bronchial epithelium sampled at the time of bronchoscopy in patients suspected of lung cancer. An 80-gene classifier was identified in a training cohort of 77 patients that distinguished smokers with and without lung cancer with a sensitivity of 80% and specificity of 84% in an independent validation cohort.

This approach was applied in the work-up of patients suspected of lung cancer, in the context of a non-diagnostic bronchoscopy, where a classifier was identified based on expression of 17 genes in bronchial epithelium. The classifier had 93% sensitivity and 53% specificity, and a negative predictive value of 94%, meaning it could be used as an adjunct when bronchoscopy is non-diagnostic to identify low-risk patients that do not need follow up procedures [79]. This classifier was prospectively validated in a larger cohort of 639 patients undergoing investigation of suspected cancer. Overall the sensitivity for the classifier was 89%, in comparison with 75% for bronchoscopy alone. Interestingly, the sensitivity of the classifier was similar for peripheral and central lesions, in contrast to bronchoscopy, where sensitivity decreased to 55% for peripheral lesions. Combining bronchoscopy and the classifier led to an overall sensitivity of 97% for diagnosis of lung cancer [80]. However, due to the highly selected nature of this population, with more than 70% developing lung cancer, it is unclear how this would perform in a screening context.

Auto-fluorescence bronchoscopy (AFB) capitalizes on the observation that the emission spectrum of bronchial mucosa under blue light changes when dysplastic or carcinomatous lesions develop [81,82]. After a degree of image processing, normal bronchial mucosa is displayed as green, in contrast to reddish brown for dysplastic/carcinomatous lesions [83,84]. In one study of 186 patients referred due to sputum atypia, an abnormal CT or CXR, patients were evaluated sequentially by white light, followed by AFB. Samples of suspicious lesions were then taken for histology. Positive histology was obtained from 32 patients with white light and AFB identified an additional 8 [85]. Several other studies have been conducted with similar methodology and were recently included in a meta-analysis. The sensitivity/specificity of AFB was 89%/64% compared with 67%/84% for white field. Thus AFB is more sensitive, but white field more specific, with bronchitis being the major confounder for AFB [84]. AFB is particularly suited to squamous cell carcinoma *in situ*, which is a relatively rare finding thereby limiting its role. However, it has been included as part of the work up in a multi-centre clinical trial for the early detection of lung cancer, the LungSEARCH study [86]. As discussed below, COPD patients were recruited to provide sputum for cytology/cytometry, and were then referred for CT/AFB if any abnormalities were detected. The full results for this study have not yet been reported, but will certainly be informative for screening in general and evaluating the place of AFB in screening.

## 5. Liquid biopsies

**Figure 1.**
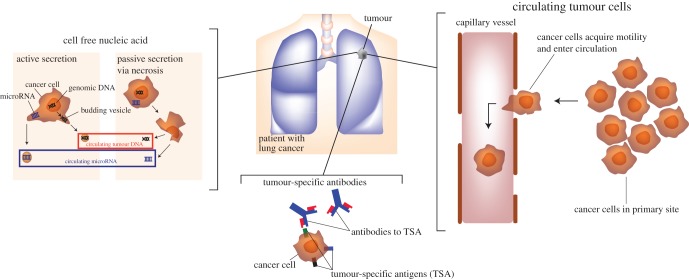
Potential targets for blood-borne biomarkers for the early detection of lung cancer.

The use of blood-borne biomarkers (so-called ‘liquid biopsies’) is beginning to gain traction for the monitoring of advanced stage lung cancers. Liquid biopsies include circulating nucleic acids, circulating proteins and circulating tumour cells (CTCs) (summarized in figure 1). While the attraction of this minimally invasive approach is clear, as a blood sample is easily repeatable and economic compared with imaging, the key issue is the sensitivity and specificity of detection for application to early diagnosis.

## 6. Circulating microRNAs in lung cancer detection

MicroRNAs (miRNAs) are essential regulators of gene expression, acting through translational inhibition or degradation of messenger RNA (mRNA) targets. Alteration in miRNA expression has been implicated in the pathogenesis of most cancers [87]. One example, let7 miRNA, is downregulated in lung cancer tissue but when overexpressed inhibits lung cancer cell line growth [88]. Conversely, over-expression of miRNA-21 has been demonstrated in both lung cancer cell lines and tissue [89].

Circulating miRNAs are present in blood from both healthy controls and patients with lung cancer. They appear to make up most of the RNA fraction in blood and are remarkably resistant to RNAse degradation. This stability means that miRNA has the potential to be used as a diagnostic biomarker. MiRNA may also enter the bloodstream in microvesicles (exosomes), secreted from platelets or phagocytic mononuclear cells. Evidence also points to exosomes originating from cancer cells [90] raising the possibility of distant signalling and preparation of niches for metastatic spread, which is a burgeoning research field [91].

Studies have looked at the association of plasma miRNA expression and lung cancer. Wozniak *et al.* [92] compared circulating plasma miRNAs in stage I–IIIA lung cancer with controls to identify a 24 miRNA panel that was differentially expressed. Another study looked at two specific miRNAs, miR944 and miR3662, in 85 healthy controls and 90 patients finding that both miRNAs were overexpressed more than fourfold relative to healthy controls in patients with NSCLC. There was no significant difference in expression between these two miRNAs between squamous cell carcinomas and adenocarcinomas [93].

Shen *et al.* [94] identified four miRNAs (mir-21, mir-126, mir-210 and mir-486–5p) in plasma that could distinguish patients with NSCLC from controls with 86% sensitivity and 97% specificity. Interestingly, this panel had a higher sensitivity for adenocarcinomas (91%) than squamous cell carcinomas (82%). The same authors also compared plasma miRNA expression between benign and malignant solitary pulmonary nodules (SPNs). Mir-21 and 210 were upregulated, and mir-486–5p was downregulated in malignant SPNs compared with benign SPNs. This allowed the authors to develop a model that had 75% sensitivity and 85% specificity for detecting malignancy from CT detected SPNs [95].

The potential for miRNA analysis to improve the effectiveness of lung cancer screening programmes has been explored. Boeri *et al.* [97] analysed miRNA expression in plasma from patients in a LDCT lung screening study [96] to identify differentially expressed miRNAs prior to development and at diagnosis of lung cancer. A panel of 15 miRNAs was identified for detection prior to diagnosis and another panel of 13 (with some overlap to the first 15) for diagnosis. These were validated on plasma samples from the MILD study [59]. Sensitivity of 80% and sensitivity of 90% was obtained for detection prior to diagnosis, while sensitivity of 75% and specificity of 100% was obtained at the time of diagnosis. Furthermore, a further panel of nine miRNAs were able to predict poor prognosis with 80% sensitivity and 100% specificity in the validation cohort [97]. These miRNAs were amalgamated into a panel of 24, which were subsequently used to generate a miRNA signature classifier (MSC), stratifying patients into low, intermediate or high risk. This tool was applied retrospectively in a blinded fashion to a larger patient cohort from the MILD study, where it had 87% sensitivity and 81% specificity. When combined with LDCT, it reduced the false-positive rate fivefold [98]. Furthermore, there was a statistically significant trend in survival from low risk to high risk in terms of 5-year survival. However, it did not perform as well as clinico-pathological staging at predicting prognosis [99]. The use of circulating MiRNAs appears to show promise for earlier detection of NSCLC, but independent, well-designed and high-powered validation studies are now required to qualify their use.

## 7. Antibodies in lung cancer detection

It is well established that the genetic aberrations involved in carcinogenesis lead to altered expression of ‘self antigens’ either through inappropriate expression of tissue specific proteins or so-called ‘neo-antigens’ (i.e. the products of non-synonymous gene mutations [100]). These tumour antigens are at the interface between the immune system and developing cancers, occurring throughout the malignant process [101] and therefore offering the possibility for exploitation as early detection biomarkers. The relationship between the immune system and cancer is complex, and the literature is weighted towards the roles of cytotoxic T cells [102]. However, it has long been recognized that the humoral immune system can be dysregulated leading to the production of autoantibodies that can be harnessed as a means for biomarker discovery [103].

There have been a number of studies that have looked for antibodies associated with the presence of lung cancer. Among the first were p53 antibodies, which are present in around 12% of lung cancer patients (including SCLC and NSCLC) [104]. Indeed, the potential that these could hold was underscored by the emergence of p53 antibodies prior to radiologically detectable lung cancer [105]. Since then several antibodies have been reported to be associated with lung cancer, and these have been amalgamated into panels for early detection. One study identified five epitopes from a phage display library of NSCLC that reacted more strongly with serum from patients with NSCLC than controls. Combining the analysis of these epitopes distinguished NSCLC from controls with 91% sensitivity and 91% specificity [106].

A panel of six antibodies (antibodies to p53, NY-ESO-1, CAGE, GBU4-5, Annexin 1 and SOX-2) was tested at the time of diagnosis of lung cancer on a cohort including both SCLC and NSCLC patients and matched controls. Sensitivity of 37% and specificity of 90% was achieved in the final validation group [107]. This panel of antibodies was tested on post-validation groups with similar results, and is now a commercially available assay with the trade name EarlyCDT-Lung [108]. The same authors used this panel of six antibodies, in addition to anti-Hu-D, in a cohort of SCLC patients. Antibodies to Annexin 1 were dropped from analysis due to no difference in levels between SCLC patients and controls. Setting specificity to 90% for the remaining panel of antibodies, sensitivity was 50%; however, these findings were not validated in an independent population [109]. The original panel was then revised to give a seven antibody panel including p53, NY-ESO-1, CAGE, GBU4-5, Hu-D, SOX-2, MAGE-A4 to improve sensitivity/specificity to 47%/90% [110]. EarlyCDT-Lung has had a limited introduction into clinical practice, where it has been used by clinicians in the work up for lung cancer in high-risk patients. The first 1699 patients (including both the six and the seven antibody panels) were included in an audit with a follow-up of 6 months and found sensitivities/specificities of 46%/83% for the six-antibody panel and 37%/91% for the seven-antibody panel [111]. A Scottish trial of 12 000 people, randomized to either EarlyCDT-Lung or usual care (i.e. no screening), aims to determine the role of this blood test in a lung cancer screening programme [112].

Antibodies to tumour-specific antigens have been applied to LDCT trials to determine their effectiveness in detecting lung cancer. To this end Trudgen *et al.* used a panel of six antibodies (antibodies to APEX1, NOLC1, PXN, Bac clone R580E16 and MT-RNR2) on serum obtained from lung cancer patients prior to diagnosis and at diagnosis, as well as controls. Using the Mayo LDCT cohort as a training group and Marty Driesler Lung Screening project as a validation cohort, sensitivity for NSCLC was 58% overall, and specificity was 43% [113]. The low sensitivities and specificities obtained in these studies illustrate the difficulty in finding consistent serological changes that are unique to lung cancer, and may limit the utility of these assays in large screening programs.

## 8. ctDNA in lung cancer detection

DNA is thought to enter plasma either passively through cell death (necrosis or apoptosis), or by active secretion from living cells. A portion of this cell free DNA in patients with cancer arises from the tumour and forms the so-called ‘circulating tumour DNA’ (ctDNA) fraction [114]. The utility of ctDNA in lung cancer was demonstrated in a study of NSCLC, where common mutations were identified to create a library for detecting mutations associated with NSCLC. Sensitivity and specificity of 85% and 96%, respectively, were obtained in a validation cohort of healthy controls and patients with NSCLC. There was also a correlation between tumour volume and amount of ctDNA within patients over time, opening the prospect of using ctDNA to monitor response to treatment. ctDNA was detectable in all late stage NSCLC cases, but only 50% of early stage cases [115].

A multinational European prospective study GENAIR obtained blood samples from healthy people at risk of cancer by means of occupational/tobacco exposure. Mutations in *TP53* and *KRAS* were detectable on average 20 months and 14 months prior to cancer diagnosis, respectively, but only in 4.6% of patients for *TP53* and 1.5% for *KRAS*. Furthermore, 3% and 0.9% controls that did not develop cancer in over 5 years of follow up were also positive for *TP53* and *KRAS* mutations [116]. In working towards developing an assay for early detection, one study evaluated mutations in *TP53* in early and late stage SCLC, comparing these with controls to get an idea of the specificity of this approach. *TP53* mutations were identified in 35% early stage and 54% late stage tumours, but these could also be identified in 11% matched controls [117].

The total amount of circulating DNA has been estimated through quantifying the human telomerase (*hTERT*) gene. Using this approach, patients with NSCLC had much higher circulating levels than age/sex/smoking matched controls [118]. A follow-on study looked at total circulating DNA in a population of patients with a significant smoking history included in an observational study for LDCT. There was no association between total amounts of circulating DNA at baseline and risk of subsequent cancer, although more circulating DNA at diagnosis was predictive of a worse prognosis [119]. Furthermore, measuring total circulating DNA is not specific for NSCLC; increased amounts of circulating DNA are also found in idiopathic pulmonary fibrosis for example [120].

The current clinical utility of ctDNA lies in the personalization of ctDNA assays based on biopsy derived genomic landscapes, and the subsequent monitoring of patient response and emergent resistance to treatment and of tumour evolution [121]. In a screening context, common lung cancer mutations such as in p53 can be used, but are also present in patients with a smoking history in the absence of lung cancer, confounding specificity [122]. Furthermore, there is growing evidence for extensive genetic mosaicism in healthy tissue, including the presence of mutations in genes that have a known role in cancer [123]. While the sensitivity of candidate gene analysis using droplet digital PCR based approaches is higher, a broader panel of genetic mutations could be more informative on tumour presence as the sensitivity of next-generation sequencing increases.

## 9. Circulating tumour cells in lung cancer detection

As aggressive cancers grow and develop, cell subpopulations acquire altered phenotypes and become motile, invading surrounding tissue and gaining access into the blood stream via a number of mechanisms including epithelial to mesenchymal transition [124], cell cooperation [125] and vasculogenic mimicry [126]. These so-called CTCs are heterogenous and are postulated to harbour the subset of cells responsible for the development of distant metastases [127]. Lending credence to this view in the lung cancer field, CTCs derived from SCLC patients have been shown to be tumourigenic in mice, forming explants that also accurately re-capitulate the response to treatment seen in the original patients [128]. There are several methods of detecting CTCs [129] and as such they have an emerging role as both a qualitative and quantitative biomarker of cancer burden.

CTCs captured via EpCAM expression and enumerated (using the CellSearch platform) are prognostic in metastatic breast [130], prostate [131] and colorectal cancers [132]. CellSearch detected CTCs are particularly prevalent in SCLC; in one study 85% of patients had detectable CTCs and greater than 50 CTCs in 7.5 ml blood CTCs was an independent prognostic biomarker [133]. However, while still prognostic (at greater than 5 CTCs in 7.5 ml blood) in NSCLC, CellSearch CTCs were detected in only 23% patients with stage III/IV disease in contrast to a size-based filtration method that yielded detectable CTCs in 80% patients in the same cohort [134]. Nonetheless, a study in NSCLC was able to increase the sensitivity of CellSearch in NSCLC by measuring CTCs from the pulmonary veins at the time of surgery in stage I–IIIA NSCLC where higher numbers of CTCs correlated with a shorter patient survival [135]. These data imply that CTCs are shed from earlier stage NSCLCs and may therefore have utility for earlier detection.

There has been interest in looking at CTCs in early stage disease/diagnosis. In a single centre study of patients referred to a thoracic centre with either a pathological diagnosis of lung cancer (SCLC and NSCLC) or high suspicion, CellSearch was used to quantify CTCs, but they were only present in 31% of patients who were subsequently diagnosed with lung cancer. However, the number of CTCs did correlate with later stage disease, in agreement with previous studies [136].

Using different methods of CTC detection is likely to yield dividends in early detection. Isolation by size of epithelial tumour cells (ISET) detected CTCs in 50% of patients with NSCLC prior to radical treatment, in contrast to 39% with CellSearch. Combining the two techniques led to detection in 69% patients [137]. The best evidence so far for CTC utility in earlier detection of lung cancer was observed using ISET alone in a population of 168 COPD patients; only the five patients with detectable CTCs developed lung cancer during the course of a 5-year follow up. This was in contrast to rest of the patients without CTCs who were lung cancer-free at the end of the follow-up period [138].

Another study used a ligand PCR method to quantify CTCs. After immune-depletion of erythrocytes and leukocytes, cells were labelled with a ligand for the folate receptor (FOLR1) conjugated to an oligonucleotide, allowing quantification by real time PCR. This approach led to detectable CTCs in 8 of 10 stage I/II NSCLC patients tested and overall a sensitivity of 82% and specificity of 93% for detection of stage I–IV NSCLC versus controls [139].

The technical hurdle for CTC analysis is the extremely rare occurrence of CTCs in even advanced late stage patients relative to the overwhelming number of blood cells in the sample. CTC heterogeneity confounds marker dependent capture, and not all CTCs are larger than blood cells, posing confounders on size-based methods. Moreover, any CTC enrichment step incurs cell loss. New approaches such as the high definition-single cell analysis platform is more suited to early detection as all cells in the sample are assessed using a flexible panel of markers and cells can be imaged and physically picked for single cell molecular analysis to confirm tumour origin [140]. The utility of CTC analysis for early detection remains to be comprehensively addressed and the question of whether survival time will be lengthened once CTCs are detectable remains unclear. Molecular profiling of ‘early’ CTCs may inform earlier intervention with personalised treatment.

## 10. Sputum analysis

As described above, initial studies of lung cancer screening with sputum cytology were disappointing. However, there is renewed interest in analysing sputum through automated cytometrics as well as new molecular techniques. One example is the multi-centre UK trial LungSEARCH, where COPD patients have been randomized to yearly sputum cytology/cytometry or no screening. Patients with positive cytology/cytometry then receive thoracic CT and AFB [86].

In one study, induced sputum from patients with asbestosis, silicosis or undergoing follow up for resected lung cancer was collected and compared with sputum from heavy smokers and healthy controls. Combining sputum cytology with quantitative image cytometry using the Cyto-Savant cytometer led to a specificity of 90% and sensitivity of 80% [141]. Another study looked at the combination of LDCT and sputum cytology in 187 high-risk asbestos-exposed patients. Although limited by small numbers, during 3 years of follow-up 18 patients developed lung cancer, of which 6 were detected by sputum and LDCT, 5 by CT alone and 1 by sputum alone [142].

MicroRNA has also been measured in sputum as a means of early detection. In one study of lung squamous cell carcinoma a panel of three miRNAs (mir-205, mir-210 and mir-708) had a diagnostic sensitivity of 72% and specificity of 95% of distinguishing patients with squamous cell carcinomas from controls [143]. A further study in lung adenocarcinoma used a panel of four miRNAs (mir-21, mir-486, mir-375 and mir-200b) had a diagnostic sensitivity of 70% and a specificity of 80% of distinguishing NSCLC (including both squamous and adenocarcinoma), although these values were more accurate when only considering lung adenocarcinomas [144]. This latter study reflects the difficulty in developing a robust panel of miRNAs that can be used on an unselected population for screening. In an attempt to combine sputum miRNA with thoracic CT, one study showed that a panel of three miRNAs (MiR-21, 31, 210) could distinguish CT detected solitary pulmonary nodules (SPNs) from malignant neoplasms with a sensitivity of 80–82% and a specificity of 86–88% [145].

There has also been interest in applying DNA mutational analysis to sputum as a means of lung cancer early detection (reviewed in [146]). Interestingly, one retrospective study comparing sputum samples taken prior to histological diagnosis of lung adenocarcinoma found that *KRAS* mutations could be detected in the sputum of 5 out of 11 patients with *KRAS* positive tumours between 1 month and 4 years prior to clinical diagnosis [147]. A prospective study in a LDCT cohort tested sputum for *KRAS* and *p53* mutations, in addition to *p16^INK4A^*, *RASSF1A* and *NORE1A* hypermethylation in 820 heavy smokers. At least one mutation or hypermethylation was present in 56 patients, of which only 1 developed lung cancer after 3 years of follow up [148].

Application of molecular and cytometric analysis to sputum has yielded interesting results, with specificities of more than 90% seen in cytometry and microRNA analysis, offering potential as adjunctive tests to reduce the false-positive rates in LDCT screening. However, their use in early detection is yet to be established.

## 11. Exhaled breath analysis

As a completely non-invasive and readily available patient sample, exhaled breath offers great potential as a screening tool. As an example in respiratory medicine, exhaled nitric oxide is now recommended by NICE as an option to help diagnose asthma [149]. There have been several interesting studies using exhaled breath as a means of lung cancer detection. Perhaps the most intriguing involved training dogs to distinguish lung and breast cancer patients from controls on the basis of volatile components (VOC) in breath samples captured on silicone oil-coated polypropylene soaked cotton wool. In a double-blinded validation cohort sensitivity and specificity were both 99% [150]. However, a more recent study with a similar design and sample size had sensitivity of 71% and specificity of 93% for canine detection of lung cancer [151]. At the least these studies provide some proof of principle that there is a detectable ‘smell print’ for lung cancer and to this end, several devices have been used to provide reproducible measurements of exhaled VOC in lung cancer [152]. Ion mobility spectrometry offers a sensitive means of detecting volatile compounds in exhaled breath and in a pilot study patients with lung cancer were readily distinguished from controls [153]. Analysis of VOC by gas chromatography mass spectrometry was used in a training cohort to look for a signature of lung cancer. In an independent cohort, including healthy controls, patients at risk of lung cancer (without a diagnosis) and patients with a tissue diagnosis, this had a sensitivity of 68% and specificity of 68% [154]. Composite measures of VOCs have also been used. One system measures changes in the oscillating frequency of quartz crystals caused by adsorption and desorption of VOCs. Lung cancer could be distinguished from healthy controls with a sensitivity of 85% and a specificity of 100% [155]. The Cyranose 320 consists of carbon black polymers that change electrical resistance in response to adsorption of VOCs. Comparing healthy controls to patients with lung cancer, a ‘smell print’ for cancer was generated in a training cohort, which had a sensitivity of 71% and specificity of 92% in an independent validation cohort [156].

One can infer from these studies that lung cancer changes the chemical composition of breath and that current technology is sufficiently sensitive to detect these changes. However, at this stage it is not clear whether similar changes can be detected prior to radiological change or in early stage disease and whether using this modality will increase the specificity of low-dose CT.

## 12. Discussion

Early detection is critically important if a significant reduction in lung cancer morbidity and mortality is to be realized. In this regard, the enormous potential of LDCT has been conclusively demonstrated by the NLST trial, which has provided a rationale for LDCT screening in high-risk ever-smokers. However, NLST selection criteria are not optimal and may exclude many patients with early lung cancer as shown in a retrospective study of patients diagnosed with stage I–II lung cancer, where just 48% met the NLST inclusion criteria for age and smoking [157]. Furthermore, one study estimated that just 26.7% of lung cancers diagnosed in the United States were covered by NLST criteria, using data from SEER (surveillance, epidemiology and end results) [158]. Improving the precision of population selection is also likely to improve screening performance further and reduce harm.

Although some patients at risk of lung cancer have other co-morbidities that could preclude surgery, new approaches such as stereotactic ablative body radiotherapy (SABR) have had promising results in treating patients with stage I lung cancer who are not fit for surgery, meaning more patients could benefit from early detection through screening [159]. It is also not clear how many CT scans patients should have over the course of their lifetime. Given that new cancers were detected at each screening interval in LDCT trials, it would seem that regular CTs are needed, but clearly there is an oncogenic risk with repeated radiation doses. A recent study estimated that 4 cancers would be induced per 10 000 patients in lung cancer screening trials [56]; however, this figure included the significant radiation doses of follow-up PET CTs performed after positive LDCT scans. Application of better risk calculators and improved imaging protocols for following patients up could aid to reduce this risk somewhat.

The caveats of LDCT include high false-positive results and the fact that late stage cancers still emerge between screening intervals. Thus there is a need for further complementary tests both to reduce the number of false positives and to work in the blind spot of LDCT to detect aggressive cancers early. Furthermore, better identification of patients at risk could reduce the number of patients requiring screening.

Several modalities have been explored in the search for biomarkers of lung cancer. Plasma microRNA and antibody assays appear to have adequate specificity to address the high false-positive rate of LDCT. Plasma microRNAs hold the most promise in this regard; with a retrospective study predicting a fivefold reduction in false-positive rates when used in conjunction with LDCT [98]. In contrast to antibody assays, plasma microRNAs also appear to have a reasonable sensitivity, but they are untested in terms of diagnosing aggressive cancers missed by LDCT. Thus there is still a great need for further tests, particularly aimed at early detection of aggressive cancers. Given that the number of CTCs correlates with a poorer prognosis, it follows that these could be harnessed as biomarkers for early aggressive disease. The understanding of early molecular events in lung tumourigenesis is increasing, with elegant and comprehensive studies in mouse models [16], which could lead to biomarker development based on detailed knowledge of early lung cancer biology. However, the relative paucity of patient relevant preclinical models of early lung cancers remains a challenge in translation of lung cancer screening.

There has been much interest in using sputum as a means of early detection from the beginning of lung cancer screening trials; however, early results in the 1970s were disappointing. With the rise of molecular biology there has been renewed interest in sputum analysis, but as yet no definitive benefit in using this modality has been identified.

The novel biomarkers discussed hold much promise for the field of early detection. However, there are some fundamental challenges that need to be considered. First, the nature of conducting biomarker research studies that are large enough to be informative is costly. Patient recruitment can also be an issue, given that lung cancer prevalence is highest in socio-economically deprived areas. Innovations that can increase participation in this demographic are vital and include community-based research studies that are geographically easier to attend.

Biomarker discovery in lung cancer can be easily confounded by the high prevalence of chronic lung disease in patients with lung cancer. To this end, biomarkers that are direct products of the tumour are likely to hold promise, but specific knowledge of the tumour in each patient is needed to fully harness this potential. This approach has therefore been successful in the TRACERx study, where specific DNA mutations identified in resected tumours were monitored through ctDNA after surgery [121], but in the context of early detection, where details of the tumour are unknown, it is altogether more difficult. A further challenge relates to the nature of novel biomarkers themselves, which at present are generally only detectable in specific assays that involve complex analyses, requiring the expertise of specialist research laboratories to interpret. Hence, as biomarkers emerge, there will be a need for more extensive work to develop these tests in a form that is reproducible in clinical laboratories and can be interpreted in community settings.

In summary, the field of lung cancer screening holds much promise. Its future success is likely to be realized in a multi-modality approach including both radiology and molecular assays. The benefit of screening is intimately related to the options available for treatment and to this end surgical resection has a proven track record. Furthermore, in patients who are not fit enough for surgery, new interventions like SABR hold promise. In the event that patients are unable to tolerate these therapies, early diagnosis holds other benefits, including the opportunity for early planning of care and engagement with cancer services at an early stage to improve quality of life.
